# Endoscopic surgery of the frontoethmoidal osteomas^☆^

**DOI:** 10.1016/j.bjorl.2017.11.005

**Published:** 2017-12-26

**Authors:** Tomasz Gotlib

**Affiliations:** Medical University of Warsaw, Department of Otolaryngology, Warsaw, Poland

Dear Editor,

I have read with the great interest the article entitled “Giant fronto-ethmoidal osteoma – selection of an optimal surgical procedure” by Maria Humeniuk-Arasiewicz et al.^1^

According to the definition used by the authors “giant osteomas” are these measuring more than 30 mm. Although suggested by the title and mentioned in “objective” section, the authors presented a review of 37 osteomas from the literature, including 12 smaller than 30 mm.

Reviewing the literature the authors selected these studies in which “authors presented operated osteomas in coronal plane”. This automatically excludes large case series, whose authors could not present graphically all the osteomas.

Three large case series of frontal sinus osteomas were ignored by the authors (total 76 cases).^2^, ^3^, ^4^ Two of them present results of endoscopic surgery.^2^, ^4^ At least a few cases from these studies should have been included in the analysis. However, these studies not only do show the examples of coronal CT of giant osteomas, but also present the current state of art of endoscopic surgery of frontal sinus osteomas, which was neglected by the authors in the discussion.

Currently frontal sinus osteomas involving the anterior and posterior frontal sinus wall, penetrating more than 2 cm above the frontal beak and lateral to the lamina papyracea, and even these totally filling the sinus (Type IV osteomas) can be safely and effectively removed endoscopically using Draf IIb, extended Draf IIb or Draf III approaches.^2^, ^3^, ^4^ The limitations of the endoscopic approach are: small anterior-posterior dimension of the frontal ostium, as well as the unfavorable shape of the posterior table and extension lateral to the mid-orbital line, which can be overcome to some extent by using an orbital transposition technique or piezosurgery.^2^, ^4^

Osteomas with large orbital or external extension, with erosion of the posterior table of the frontal sinus can be challenging to manage endoscopically, and may require an additional external approach.^2^, ^3^

In contrast, the vast majority of the osteomas of the ethmoidal sinuses only partially involving the frontal ostium (regardless the size) can be safely removed endoscopically without resection of the inferior nasal turbinate and creation of a septal perforation as presented by the authors. This can be achieved by drilling the central part of the tumor and leaving residual peripheral egg shell fragments, which are broken and removed at the end of the procedure.^5^

However, it is difficult not to agree with the author's conclusion that the surgical approach depends on the past experience of the surgeon, available equipment and knowledge of different surgical techniques.

## Conflicts of interest

The author declares no conflicts of interest.
